# Generalised Gingival Fibromatosis and Hypertrichosis: A Rare Case of Syndromic Presentation

**DOI:** 10.7759/cureus.100988

**Published:** 2026-01-07

**Authors:** Dilip Goswami, Reshu Rai, Prachi Jain, Parivraj Priyam Goswami

**Affiliations:** 1 Periodontics and Oral Implantology, Regional Dental College, Guwahati, IND; 2 Medicine, Saveetha Medical College, Chennai, IND

**Keywords:** androgen, fibroepithelial hyperplasia, gingival fibromatosis, gingivectomy, hypertrichosis

## Abstract

Gingival fibromatosis is a benign oral condition characterised by a slow progressive, non-haemorrhagic, non-painful, local or generalised enlargement of the gingival tissues. Gingival fibromatosis may have a genetic basis and is frequently associated with systemic or extraoral findings. Hypertrichosis is a clinical condition characterised by excessive hair growth without androgenic stimulation. The present case report describes a case of a 16-year-old girl who presented with severe generalised gingival fibromatosis along with hypertrichosis, which was treated by gingivectomy, and uneventful postoperative healing was observed during the eight-month follow-up period.

## Introduction

Fibromatosis is defined as the simultaneous development of many fibrous, encapsulated connective tissue tumours. Gingival fibromatosis is defined as an infiltrating fibroblastic proliferation showing none of the features of an inflammatory response and no features of unequivocal neoplasia. It can be hereditary or idiopathic. It has also been designated by the terms gingivomatosis, elephantiasis, familial elephantiasis, juvenile hyaline fibromatosis, congenital familial fibromatosis, idiopathic fibromatosis, idiopathic gingival fibromatosis, hereditary gingival hyperplasia, and hereditary gingival fibromatosis [1-3]. The condition may appear as a generalised process involving both maxillary and mandibular arches, or it may be incomplete, involving only localised areas of either arch [4]. The disease presentation is usually between the ages of 10 and 35 years. The clinical presentation involves a firm, leathery, and minutely pebbled gingival enlargement that is pink with brown melanin pigmentation. When severe, the gingival tissue becomes bulbous, often almost completely covering the teeth and projecting significantly into the oral vestibule, causing the jaws to appear distorted. Inflammatory changes at the gingival margin are frequently observed as a secondary feature. The term "gingival fibromatosis with hypertrichosis (GFH) syndrome" describes the combination of gingival overgrowth with abnormal hair growth [5]. The historical context includes descriptive terms such as "wolf men" and "bear woman" [6] for individuals with severe hypertrichosis. Hypertrichosis is defined as a clinical state characterised by excessive, non-androgenic hair growth [7]. When no underlying aetiology for this excessive hair growth is identifiable, the condition is termed primary hypertrichosis. This primary form is systematically classified based on the age of onset, congenital or acquired, and the distribution of the hair growth, generalised or localised. For cases presenting with extensive overgrowth volume, a repositioned flap procedure is clinically indicated instead of gingivectomy. This approach is based on the need to prevent undesirable connective tissue exposure while simultaneously achieving elimination of pseudo-pockets.

## Case presentation

**Figure 1:**
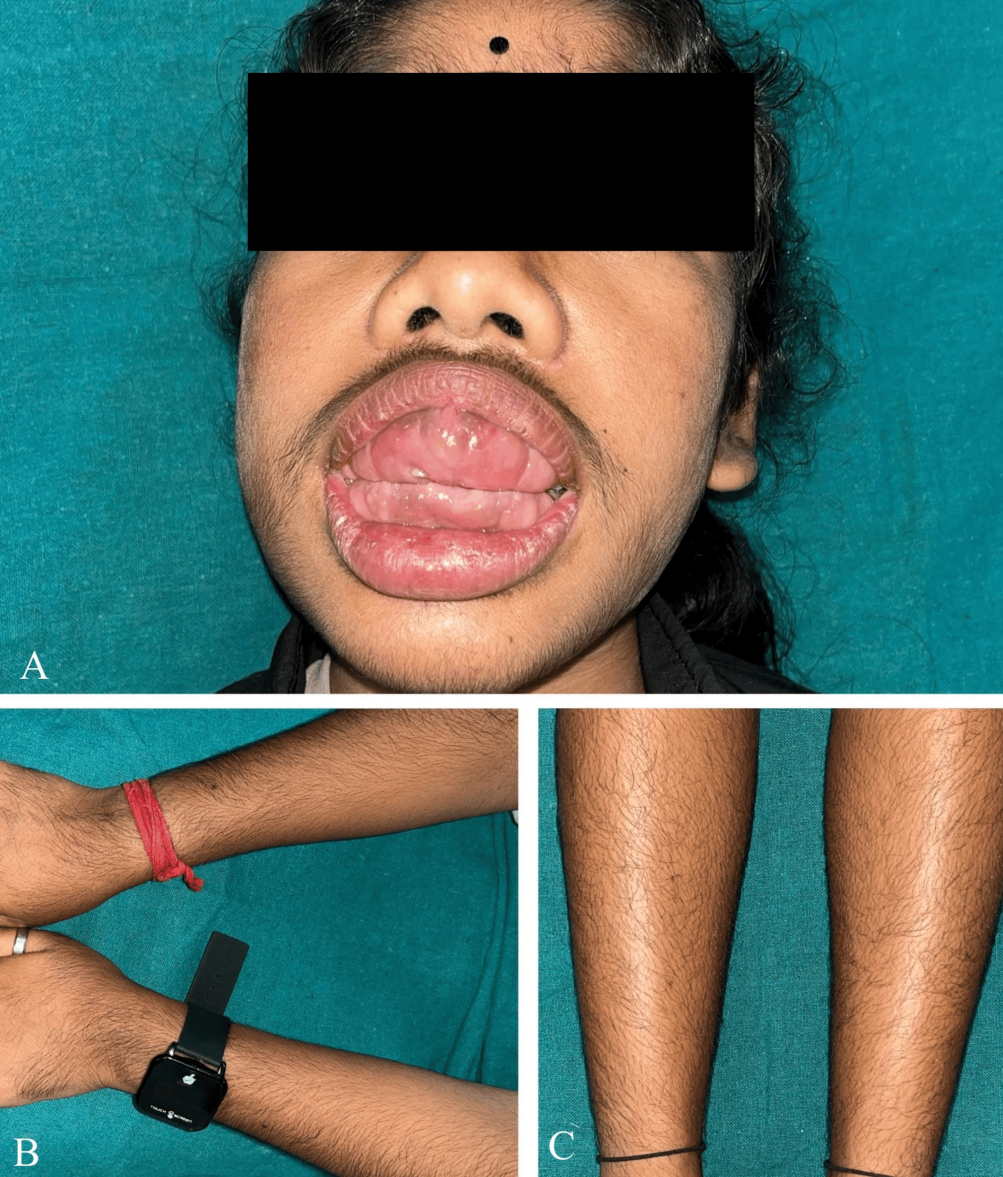
Clinical presentation of gingival fibromatosis with hypertrichosis (GFH) syndrome. (A) Extra-oral view showing marked facial hirsutism and severe diffuse gingival enlargement. (B and C) Views highlighting the generalized hypertrichosis (excessive hair growth) on the upper and lower extremities. Images reproduced with written informed consent obtained from the patient for publication.

A 16-year-old female from Assam presented to the Outpatient Department (OPD) of Periodontics and Oral Implantology at Regional Dental College, Guwahati, accompanied by her parents. Her primary complaint was significant gum enlargement, which interfered with her ability to eat and speak. This condition caused her considerable self-consciousness, leading her to discontinue her schooling and wear a mask to avoid social embarrassment. Her medical history was unremarkable for any past or present systemic illnesses. The mother reported a normal, full-term pregnancy without any complications and denied any intake of medications or chemicals during that period. On clinical examination, the patient was mentally alert, of short stature, and had a normal weight for her age. She exhibited a hoarse voice, incompetent lips, and a tongue-thrusting habit. The patient displayed generalised excessive hair growth (hypertrichosis), including coarse facial hair and bushy eyebrows that met at the midline (Figure 1). Facial inspection also noted a wide and depressed nasal bridge.

**Figure 2:**
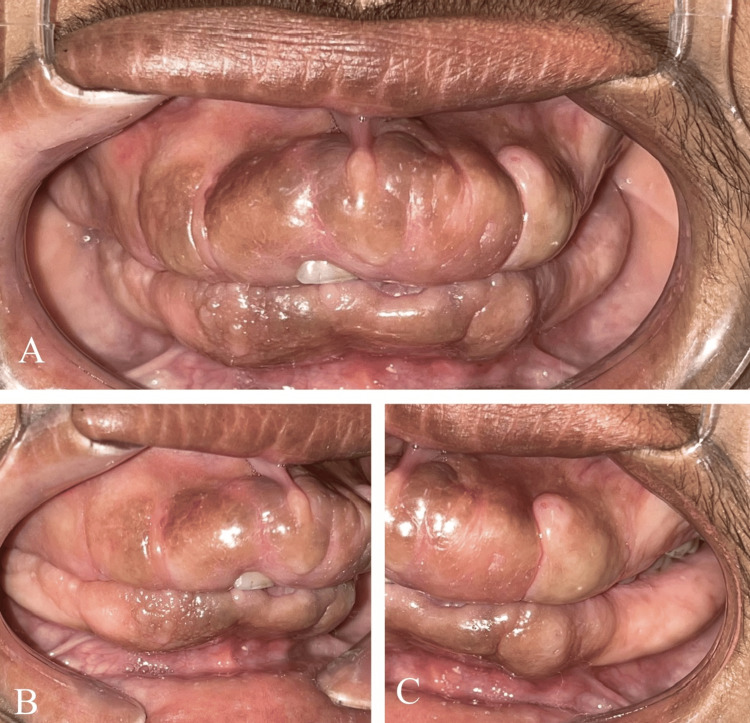
Intra-oral clinical findings. (A) Maxillary and mandibular arches showing Grade III diffuse gingival enlargement, pale pink color, firm consistency, and superimposed melanin pigmentation. (B and C): Different views illustrating the extent of the enlargement, which covers most of the tooth surfaces.

The intraoral finding was prominent, showing diffuse Grade III gingival enlargement affecting all teeth (Figure 2). The gingival tissue was pale pink with melanin pigmentation, firm, and fibrous. It was painless and extended to cover all tooth surfaces, except the occlusal tables of some posterior molars. The tissue did not bleed upon gentle probing. Pseudopockets ranging from 5 to 10 mm were measured. The patient's mother reported that the hyperplasia had developed slowly, coinciding with the eruption of the permanent teeth, eventually affecting the entire dentition. Routine haematological, urine, and hormonal investigations were performed, with all results falling within the physiological range. Due to the patient's poor socioeconomic status and associated financial constraints, the planned genetic mapping study had to be deferred.

**Figure 3:**
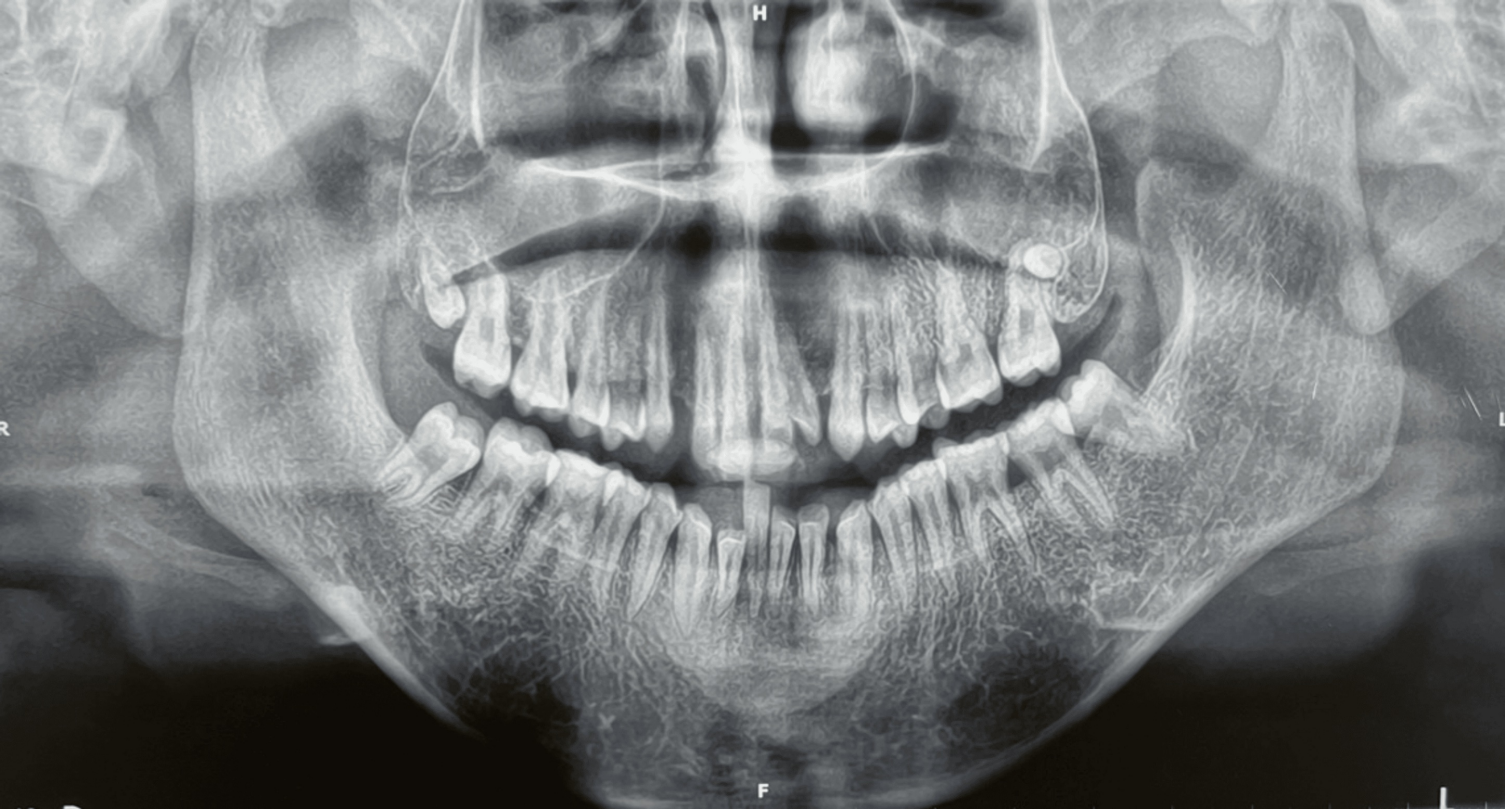
Pre-operative orthopantomogram (OPG) panoramic radiograph showing the presence of all permanent teeth along with developing upper third molars and no evidence of alveolar bone loss.

An orthopantomogram (OPG) was performed, which revealed the presence of all permanent teeth. The upper third molars were not fully formed. There was no radiographic evidence of alveolar bone loss (Figure 3).

Treatment

**Figure 4:**
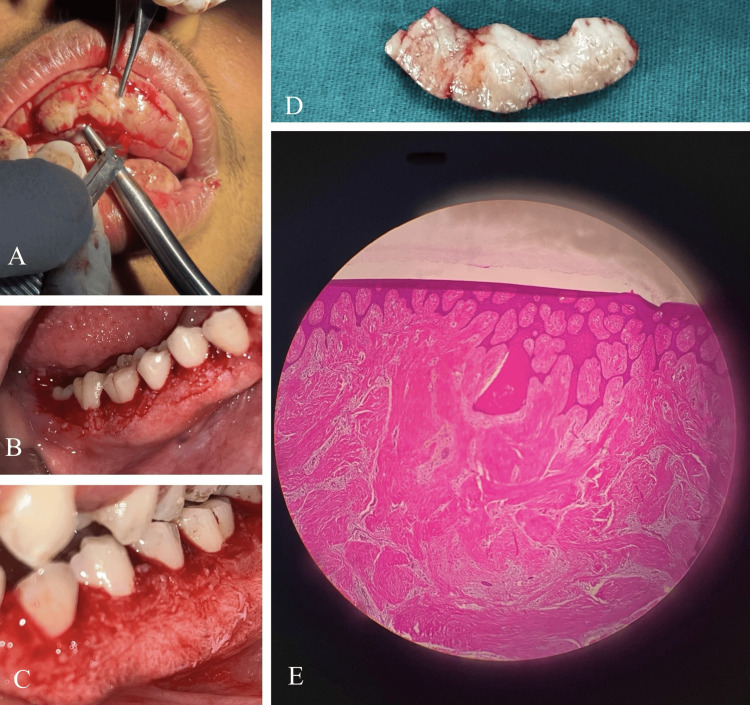
Surgical and histopathological view. (A) Intra-operative view showing the surgical excision of enlarged tissue (gingivectomy). (B and C) Post-surgical views of the lower arch demonstrating visible dentition after tissue removal. (D) Excised tissue sample sent for histopathological examination. (E) Microscopic view (H&E stain, approximately 10x magnification) showing fibroepithelial hyperplasia with dense collagen bundles and long, slender rete pegs.

The scheduled treatment plan was explained to the patient, and written consent was obtained for undergoing surgery. Phase I therapy was completed, and the patient was educated and motivated regarding the necessary oral hygiene measures to be routinely followed. Chlorhexidine (0.2% W/V) mouthwash was prescribed, and the patient was recalled after eight weeks. At the recall visit, all clinical parameters were again recorded, and necessary investigations, including complete blood count (CBC), prothrombin time or international normalised ratio (PT/INR), bleeding time (BT), and clotting time (CT), were performed for the planned surgical procedure. Gingivectomy of the enlarged tissues was performed in six separate phases under local anaesthesia, along with maxillary labial frenectomy. A long external bevel incision was meticulously made to achieve knife-edge margins (Figure 4A-4C). Samples of the excised tissues were sent for histopathological examination. The patient was recalled after each surgical phase, and uneventful healing was observed following surgery, with a complete set of visible dentition. The histopathological report of the excised tissues revealed non-dysplastic, proliferative keratinised as well as para-keratinised stratified squamous epithelium with long and slender rete pegs within the underlying connective tissue stroma. The connective tissue stroma was fibrous, with parallelly arranged collagenous bundles. Microscopic findings were suggestive of fibroepithelial hyperplasia (Figure 4D, 4E).

**Figure 5:**
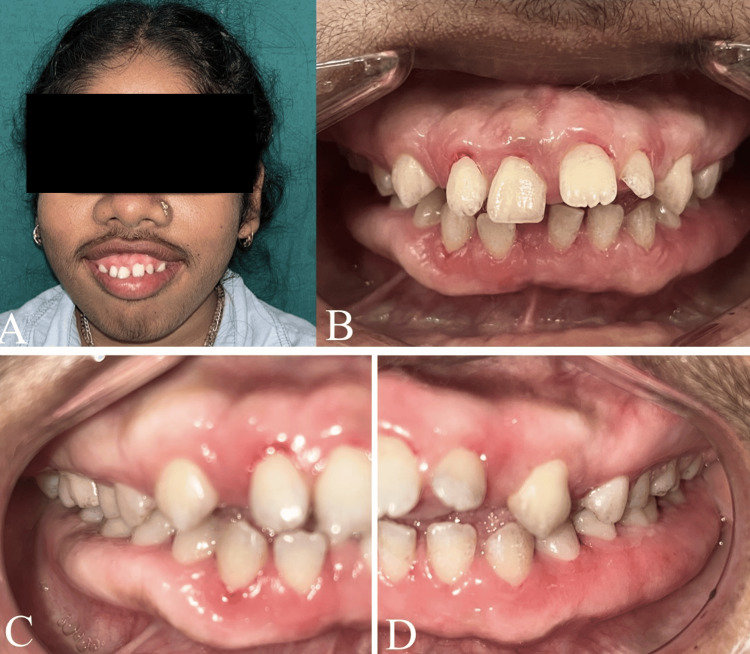
Post-surgical clinical outcome. (A) Extra-oral view showing improved lip competency and aesthetic profile post-surgery. (B) Frontal view of the dentition post-gingivectomy showing visible clinical crowns and reduced gingival margin. (C and D) Detailed intra-oral views of the maxillary and mandibular arches demonstrating complete exposure of the clinical crowns with healthy gingival tissue. Images reproduced with written informed consent obtained from the patient for publication.

The staged conventional gingivectomy performed under local anesthesia successfully resolved the patient's functional and aesthetic issues, effectively minimizing discomfort and enhancing psychological well-being (Figure 5).

## Discussion

The present clinical case highlights the presentation and management of a rare, non-inflammatory condition, gingival fibromatosis with concomitant hypertrichosis. As outlined in the literature, this co-occurrence is often a syndromic presentation, with historical references dating back to the 17th century by Bartholin (1654) and later descriptions by Humphry (1886) [6] and Byars and Sarnat (1945) [2]. The co-occurrence of gingival fibromatosis with hypertrichosis is often associated with specific syndromes [7]. A thorough physical and family history examination is critical to differentiate between isolated and syndromic forms. The pathogenesis of this condition is thought to involve an imbalance in the extracellular matrix. As reported by Coletta et al. (1999) [8], there is an alteration in the expression of Matrix Metalloproteinase-1 (MMP-1) and Matrix Metalloproteinase-2 (MMP-2), which are crucial for collagen degradation. This imbalance, mediated by autocrine stimulation of transforming growth factor-beta (TGF-beta), results in over-accumulation of collagen, leading to the characteristic fibrous overgrowth seen in this case. The absence of an inflammatory response further distinguishes this condition from other forms of gingival enlargement, such as those induced by plaque or drugs.

The management of severe gingival fibromatosis is primarily surgical [9-11], as the condition does not resolve spontaneously. The staged surgical approach of conventional gingivectomy under local anaesthesia proved effective in this case, addressing the patient’s functional and aesthetic concerns while minimising discomfort and promoting psychological well-being. However, the literature indicates a high rate of recurrence, which necessitates long-term follow-up and rigorous oral hygiene maintenance to sustain the clinical improvement.

## Conclusions

This case report provides a rare syndromic presentation of gingival fibromatosis with hypertrichosis, reinforcing the clinical and histopathological findings described in the literature. The association of hypertrichosis with a coarse facies can further worsen physical appearance. Thus, management of this case requires a multidisciplinary approach. While gingivectomy remains the primary treatment for the oral condition, orthodontic correction of malaligned teeth and management of the associated hypertrichosis require long-term planning tailored to the patient’s psychological and social needs. The high risk of recurrence in gingival fibromatosis makes regular dental follow-up mandatory, while the lifelong nature of hypertrichosis necessitates ongoing cosmetic management.
